# A pilot study to implement artificial intelligence-enabled, point-of-care obstetric ultrasound for gestational age estimation in Zambia: An evaluation protocol

**DOI:** 10.1371/journal.pone.0357962

**Published:** 2026-09-11

**Authors:** Erika Gazzetta, Tulani Francis L. Matenga, Stephanie Martin, Nelly Mandona, Rassil Barada, Modesta Chileshe, Elizabeth Stringer, Manze Chinyama, Beene Chembo, Harmony Chi, Angel Mwiche, Selia Ng’anjo, Jeffrey S.A. Stringer, Benjamin H. Chi, Margaret Kasaro

**Affiliations:** 1 Department of Obstetrics and Gynecology, University of North Carolina School of Medicine, Chapel Hill, North Carolina, United States of America; 2 Department of Health Promotion and Education, School of Public Health, University of Zambia, Lusaka, Zambia; 3 UNC Global Projects Zambia, LLC, Lusaka, Zambia; 4 Department of Nutrition, Gillings School of Global Public Health, University of North Carolina at Chapel Hill, Chapel Hill, North Carolina, United States of America; 5 Department of Public Health, Ministry of Health, Lusaka, Zambia; 6 Department of Obstetrics and Gynaecology, Women and Newborn Hospital, University Teaching Hospital, Lusaka, Zambia; University of Pretoria, SOUTH AFRICA

## Abstract

**Background:**

Ultrasound is essential for accurate pregnancy dating, but its implementation in low- and middle-income countries is hindered by cost, infrastructure, and training barriers. The implementation of point-of-care ultrasound (POCUS) with artificial intelligence (AI) technology to accurately estimate gestational age can potentially address these barriers. We describe a protocol to evaluate the acceptability, feasibility, and fidelity of integrating AI-enabled POCUS for gestational age dating into routine antenatal care (ANC) in Zambia’s Lusaka Province.

**Methods:**

PIKABU (Piloting Integration, Knowledge and Acceptability of Baby Ultrasounds) is a multi-year pilot program to introduce and maintain AI-enabled POCUS in six ANC facilities across three districts. To evaluate these activities, we designed a prospective, mixed methods evaluation to assess acceptability, feasibility, and fidelity. Informed by the Consolidated Framework for Implementation Research, the evaluation comprises six separate components: focus-group discussions, in-depth interviews, patient register reviews, time motion studies, implementation strategy assessments, and patient exit surveys. Participants include patients, community members, and healthcare providers. By collecting baseline and follow-up data every four months, we are able to measure these outcomes in longitudinal fashion.

**Discussion:**

Integrating portable, AI-enabled POCUS into routine ANC can improve gestational age dating and improve maternal health services in resource limited settings. Through its assessment of the acceptability, feasibility, and fidelity, this study provides novel insights about service implementation. Our findings are expected to inform policy and programs considering AI-enabled POCUS and support broader adoption across a range of healthcare settings.

## Introduction

Ultrasound services are a cornerstone of antenatal care (ANC) and play an important role in estimating gestational age [1]. Correct pregnancy dating is required for gestational age-dependent prenatal interventions, diagnosing preterm birth and understanding causes of adverse pregnancy outcomes such as intrauterine fetal growth restriction and stillbirth [2]. In Zambia, the majority of ANC is provided by midwives who, despite reporting a desire to include basic ultrasound education as a component of midwifery training, largely do not perform ultrasounds [3,4]. The implementation of routine ultrasound in low- and middle-income countries (LMICs) faces multiple barriers. Traditional instruments are costly, large, difficult to transport and require a reliable power source, routine maintenance and quality control [5–7]. Training providers to perform even a basic scan requires a substantial time investment and, for widespread scale up. This means either training existing overwhelmed clinicians or hiring new people to provide the ultrasounds. Additional reported challenges to scaling up services include a lack of standardized implementation protocols, high turnover of health facility staff, damaged or ineffective probes in hot climates and unstable electricity [8,9]. Health-seeking behaviors—including late presentation to the first ANC visit—can also hinder the accurate assessment of gestational age of pregnant women.

Promising advances may address these obstacles. Portable, chargeable, point-of-care ultrasound (POCUS) probes now permit assessments across a range of clinical settings. Further, artificial intelligence (AI) algorithms—embedded within app-based tools—can interpret ultrasound images, even in blind transabdominal sweeps [10–12]. In diagnostic accuracy studies, for example, novice users of an AI-enabled POCUS tool for pregnancy dating produced gestational age (GA) estimates as accurately as expert sonographers obtaining standard biometry measurements [13]. Despite the promise of such technologies, however, full-scale adoption cannot be assumed. Pilot programs play an important role for such services, identifying and addressing barriers to and facilitators to implementation. Through this evaluation protocol, we seek to assess the acceptability, feasibility, and fidelity of antenatal POCUS—paired with an AI-enabled GA tool—to improve pregnancy dating across six facilities in Zambia’s Lusaka Province.

## Methods

### Pilot program overview

PIKABU (Piloting Integration, Knowledge and Acceptability of Baby Ultrasounds) was a pilot program to implement ultrasound services within ANC in Zambia. The primary goal is to improve GA estimation, with the assumption that more accurate dating will have downstream impact on clinical decision-making for the pregnant woman and her newborn infant. The intervention comprises two parts: (1) a portable POCUS probe paired with tablet computers, and (2) an AI-enabled application tool that provides estimates of current GA and estimated due date. The GA tool has been validated through diagnostic accuracy studies, including data from Zambian participants [13]. It was approved by the US Food and Drug Administration in March 2026 as an integrated POCUS probe and software package from Butterfly Network, Inc. (Burlington, MA, USA) [14]. Combined, this approach allows for standardized blind sweeps of the gravid abdomen that can be performed by non-expert users. Both components were introduced together, integrated into ANC and in settings where such technology is limited. For the sake of brevity, we refer to this combined package (i.e., POCUS probe and GA tool) as *AI-enabled POCUS* in the remainder of this report.

AI-enabled POCUS has been integrated into maternal and child healthcare in partnership with the Zambian Ministry of Health. The implementation of services is described elsewhere [15]. Selected ANC clinics did not have prior experience with POCUS devices, but instead relied on referral mechanisms to other units and facilities. PIKABU brought in resources for training, equipment (i.e., POCUS probes), clinical oversight and supervision, information and communications technology technical support (including data bundles), and facility-based supplies. Due to the simplified nature of data capture—including the use of blind sweeps for GA determination—and the limited number of trained sonographers in our setting, we primarily relied on non-expert users (e.g., nurses, midwives, clinical officers). These individuals underwent a process that included didactic training, hands-on simulations, and supervised ultrasounds in the clinical setting [16]. They were trained to visualize fetal heart rate using the POCUS probe (thus establishing viability) and to perform standard blind sweeps to determine GA. To guide ANC, the estimated due date from AI-enabled POCUS was reported to the patient and documented in her antenatal card and on facility registers.

### Evaluation component

In this report, we focus on the prospective evaluation protocol embedded within the PIKABU initiative, designed to evaluate the acceptability, feasibility, and fidelity of integrated AI-enabled POCUS services within ANC. The overarching goal is to provide empirical implementation data to the Zambia Ministry of Health, provincial and district health officers, and health care facilities and inform similar implementation efforts in the country and region.

### Design

This protocol utilizes convergent parallel mixed methods design, where quantitative and qualitative data are collected simultaneously at intermittent evaluation periods. The evaluation design is informed by the Consolidated Framework for Implementation Research (CFIR) 2.0 [17]. We selected this determinant framework to better understand the facilitators and barriers to implementation [18].

**Fig 1:**
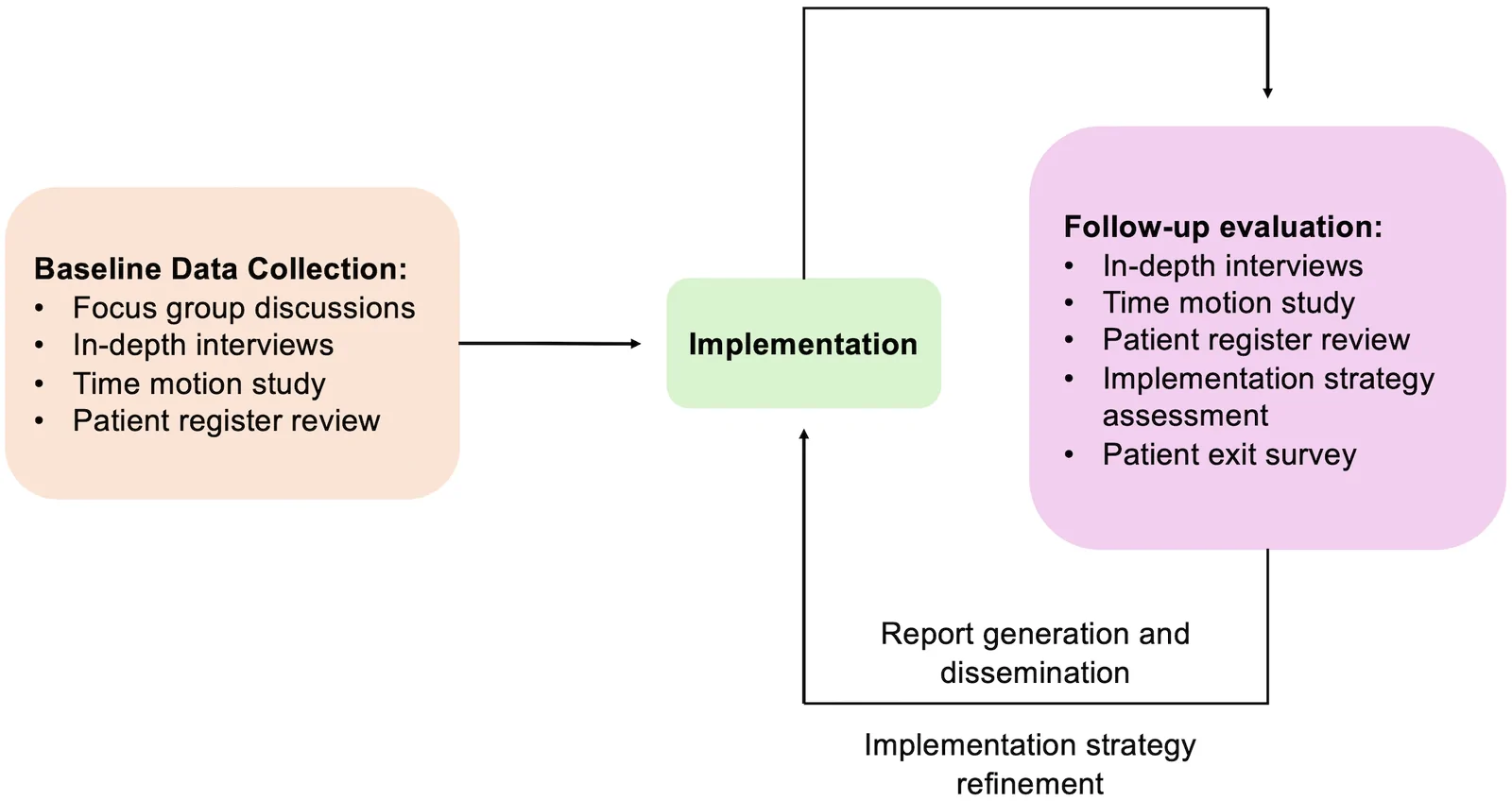
PIKABU program evaluation design.

We rely on a multi-pronged evaluation strategy to assess specific implementation outcomes: acceptability, feasibility, and fidelity of implementation. Baseline evaluation activities are conducted to understand pre-existing clinical practices, patient-seeking behaviors, and the anticipated patient and provider response to AI-enabled POCUS within routine ANC services. Following the program launch at the facility, follow-up evaluation activities occur on a recurring basis, approximately every four months. Through this repeated, longitudinal approach (Fig 1), facilities use empiric data to drive an iterative implementation process, one that best fits their facility’s context and needs. Data collection began in June 2024 and is expected to last approximately two years.

### Outcomes

Although AI-enabled POCUS is supported by a rigorous evidence base, the ability to introduce and maintain such services in public health settings is yet unknown, particularly in resource-constrained African settings where the need is great. We focus on three main outcome domains: (1) acceptability—the perception that the innovation is agreeable, palatable, or satisfactory; (2) feasibility—the extent to which the innovation can be successfully carried out within a given setting; and (3) fidelity—the degree to which the innovation was implemented as it was planned [19]. These outcomes focus on the patient and provider (i.e., acceptability) and health system (i.e., feasibility and fidelity) alike. We describe the outcome domains and specific measures in Table 1, alongside specific evaluation procedures. These outcomes are well-aligned with the objectives of the PIKABU pilot program, which was designed to provide real world data to inform broader implementation in Zambia and regionally.

**Table 1:** PIKABU outcome domains, associated evaluation components, and measures.

Outcome domain	Evaluation component	Population	Measure
Acceptability	Focus group discussions	Antenatal patients, community members	Perceptions about the potential integration of AI-enabled POCUS into antenatal care, including appropriateness, cultural acceptability, trustworthiness, and benefit to health and wellbeing
	In-depth interviews	Healthcare providers	Perceptions about the appropriateness, clinical usefulness, trustworthiness, and potential benefit for improving care of AI-enabled POCUS. Willingness to continue using and supporting the intervention package
	Patient exit surveys	Antenatal patients	Perceptions about AI-enabled POCUS, recommendations for changing POCUS experience
Feasibility	In-depth interviews	Healthcare providers	Perceptions about AI-enabled POCUS implementation, including antenatal care integration strategies and facility constraints (e.g., available time, staffing, infrastructure, equipment, training, supervision, referral systems, and supply chain processes)
	Time-motion study	Antenatal patients	Time spent for AI-enabled POCUS, compared to other parts of the antenatal care visit
	Patient register review	Antenatal patients	Number of AI-enabled POCUS performed in the two weeks prior to evaluation period
Fidelity	Patient exit surveys	Antenatal patients	Provider explained reason for and results of ultrasound. Patient understood ultrasound results
	Implementation strategy assessment	Healthcare provider	Ultrasound components communicated to patients (i.e., estimated due date, gestational age, fetal heart rate). Ultrasound results documented (antenatal registry, antenatal card).

### Evaluation procedures

The evaluation is made up of six evaluation procedures—collecting both qualitative and quantitative data—to identify barriers and facilitators, develop implementation strategies and monitor implementation processes (Table 2) [20]. Each procedure is described in detail below.

**Table 2:** Evaluation procedures and their associated data type and frequency of data collection.

Evaluation procedures	Data type	Frequency	
		Baseline	Follow-up*
Focus group discussions	Qualitative	•	
In-depth interviews	Qualitative	•	•
Patient register reviews	Quantitative	•	•
Time-motion studies	Qualitative & Quantitative	•	•
Implementation strategy assessments	Qualitative & Quantitative		•
Patient exit surveys	Quantitative		•

* Follow-up evaluation is a recurring process taking place approximately every 4 months.

Focus group discussions (FGDs) are designed to gather information relating to applicable CFIR 2.0 constructs (Table 3). They are conducted at baseline with three participant populations per facility (i.e., pregnant patients, male community members, female community members). Basic participant demographic information is collected. After a brief presentation of the portable AI-enabled POCUS, the moderator follows a semi-structured guide to assess the participants’ perceptions and responses to the innovation. FGDs are conducted in Nyanja (local language used in the area), recorded, transcribed and translated into English. Transcripts are analyzed thematically with a 4-step approach: 1) reading and summarizing transcripts to document preliminary topics, 2) hybrid coding using a combination of deductive codes based on CFIR constructs and inductive codes drawn from emerging data in the transcripts [21], 3) data reduction to identify principal subthemes reflecting finer distinctions in coded transcripts and create an inventory of what is related to each code, and 4) interpretation by making connections between themes and dissemination.

**Table 3:** Consolidated Framework for Implementation Research domains and constructs associated with components of the PIKABU evaluation.

		Assessed in:			
**Domain**	**Construct and definitions**	**Focus group discussions**	**In-depth interviews**	**Patient exit surveys**	**Implementation strategy assessments**
Innovation AI-enabled POCUS	Evidence base: perceptions of the quality and validity of evidence supporting the use of AI-enabled ultrasounds	•	•		
	Relative advantage: perceptions of how the AI-enabled ultrasounds compare to current practice	•	•		
	Adaptation: modifications or refinements that can be made to adapt AI-enable ultrasounds to local context and/or needs	•			
	Complexity: perceptions of the difficulty of the delivering AI-enabled ultrasounds	•	•		
	Design: perceptions of the design, assembly and presentation of the AI-enabled ultrasound devices and implementation strategy		•		
Outer setting Community, healthcare system	Local attitudes: How sociocultural values and beliefs encourage the Zambian antenatal care system to support the delivery of AI-enabled ultrasounds	•			
	Local conditions: The economic, environmental, political and/or technological conditions enabling the Zambian health care system to support the delivery of AI-enabled ultrasounds	•			
	Partnerships & connections: The degree to which the antenatal care system is networked with external entities such as referral networks		•		
Inner Setting Antenatal care facilities	Structural characteristics: How the infrastructure components of the ANC facilities support performance of the facilities to implement AI-enabled ultrasounds		•		
	Culture: The shared values, beliefs and norms across clinics and their impact on AI-enabled ultrasound implementation		•		
	Communications: The formal and informal information sharing practicing within and across ANC facilities and their impact on AI-enabled ultrasound implementation		•		•
	Compatibility: The degree to which the delivery of AI-enabled ultrasound delivery fits with the facility workflow, systems, and processes		•		•
	Available resources: The resources available to implement AI-enabled ultrasound		•		
	Access to knowledge & information: The degree to which guidance and training are accessible to aid in implementation of AI-enabled ultrasound				•
Individuals Recipients and deliverers of AI-enabled POCUS	Need: How delivery of AI-enabled ultrasounds addresses needs of the recipients	•		•	
	Motivation: The perceived acceptance of AI-enabled ultrasounds	•		•	
	Capability: The degree to which ANC facility staff are empowered with competence, knowledge and skills to deliver AI-enabled ultrasounds		•		
Implementation Process Activities and strategies of implementation	Assessing needs: The priorities, preferences, and needs of ANC facility staff regarding AI-enabled ultrasound implementation		•		
	Assessing context: The identification and appraisal of barriers and facilitators to the implementation of AI-enabled ultrasounds		•		

AI: artificial intelligence, POCUS: point-of-care ultrasound.

In-depth interviews (IDIs) are designed to gather information relating to applicable CFIR 2.0 constructs (Table 3). They are conducted at baseline and each follow-up evaluation among healthcare providers. Basic participant demographic information is collected. The moderator follows a semi-structured guide to assess provider perceptions (i.e., acceptability) about AI-enabled POCUS in their facilities, their perceived feasibility of the implementation strategy and anticipated and/or actual barriers and recommendations. IDIs are conducted in English. A rapid qualitative analysis approach is employed. Framed by CFIR constructs and the interview guides, domains are identified and organized into an interview summary template. Each IDI recording is listened to, summarized, and an IDI summary template completed. Data from each summary template is compiled into a matrix for rapid thematic analysis where a similar 4-step qualitative analysis approach (as described above) is employed.

Patient register reviews are designed to gather information of patients presenting for ANC and those who are referred to other healthcare facilities. They are conducted at baseline and each follow-up evaluation. Each facility maintains an in-house ANC and referral register where patient and referral information are documented and stored. All records of patients presenting for ANC and those receiving a referral over the course of a consistent assessment period will be reviewed and data extracted. Information such as the date of service, gestational age at booking visit, and reason for referral will be collected. The data collected with each evaluation are descriptively analyzed and the presence of temporal trends assessed.

Time motion studies (TMS) examine the duration, location and clinical procedures that occur at each step of an antenatal visit at each facility. They are designed to characterize the patient clinical encounter throughout the ANC visit including wait time and clinical flow. TMS are conducted at baseline and each subsequent evaluation period. Pregnant women who are expected to receive AI-enabled POCUS at the visit are selected to participate via convenience sampling. Study staff shadow the patient throughout their visit and document each location the patient encounters, the procedures conducted at each location and the duration for each procedure, including provision of AI-enabled POCUS. The amount of time spent providing ultrasound and other pertinent measures (i.e., time spent receiving specific procedures, time spent waiting, total visit time) are collected and analyzed descriptively and over time.

Implementation strategy assessments (ISAs) are designed to document and assess the performance of implementation strategies at various facilities. They include a structured discussion with the facility’s manager (typically a head nurse who oversees daily clinical activities and staffing) to reflect on operational details at the facility, including educating patients about AI-enabled POCUS, integrating ultrasound into clinic flow, explaining ultrasound results, and documenting findings. Study staff also observe clinical and operational practices of the facility as they relate to the provision of the AI-enabled POCUS, using a standard study checklist of key indicators describing clinical care. A qualitative analytic approach is used to assess similarities and disparities in implementation strategies across facilities. Quantitative data are descriptively analyzed to assess performance of various components of the implementation strategy.

Patient exit surveys are structured surveys gathering information regarding patient experiences with AI-enabled POCUS. They are designed to understand the acceptability of the innovation and implementation strategy from the perspective of the innovation recipients, the patients. Conducted at each follow-up evaluation, we collect quantitative data to describe the experience of patients at each facility

At the end of each cycle, a site summary is shared with facility leadership and PIKABU support staff, based on several of the evaluation component noted above. This includes quantitative data from the patient register review and ISAs. The intent of these summaries is to highlight existing gaps and provide a basis for problem-solving and ongoing monitoring.

### Study participants and sample size

Table 4 describes each of the evaluation procedures and its corresponding participants, eligibility criteria and sample sizes. Sample sizes of qualitative evaluation processes were designed to maximize thematic saturation within certain operational considerations [22,23]. For all evaluation procedures, individuals must be 18 years. Potential participants are assessed for eligibility and enrolled following provision of informed consent.

**Table 4:** Description of participants, eligibility criteria, and anticpated sample size for each PIKABU evaluation component.

Evaluation procedure	Participants	Eligibility criteria	Sample size (each evaluation cycle & facility)
Focus group discussion	Patients Male community members Female community members	Patients: seeking ANC at the facility Community members: have a direct connection to the patient (i.e., husband, father, mother, sister)	4-8 participants per FGD 3 FGDs held at each facility
In-depth interview	Healthcare providers	Involved with AI-enabled ultrasound provision either directly (midwife) or indirectly (serving in a managerial position)	2-8
Patient register review	Patients presenting at first antenatal visit	Patient and referral information must be documented in the facility-specific ANC and referral registers.	All documented patients over the course of the assessment period
Time-motion study	Patients presenting for ANC	Patient is attending their first ANC visit	5-20
Implementation strategy assessment	Discussion: facility stakeholder Observation: healthcare providers	Facility stakeholder: involved in clinical decision making ANC staff: providing AI-enabled ultrasounds	10-20
Patient exit survey	Patients	Recipients of an AI-enabled ultrasound	15-30

### Study setting

The PIKABU program is being implemented in healthcare facilities providing ANC in Zambia. Within the Lusaka province, three districts were selected: Chongwe, Kafue, and Lusaka. In consultation with the Ministry of Health, a comprehensive site-selection process was employed to identify sites where implementation was determined to be feasible and accepted by healthcare providers and district-level policymakers. Two facilities from each district were selected to represent a diverse array of facilities (Table 5) as we expect the evaluation of program implementation in multiple facility types and settings will enhance the generalizability of implementation evaluation results. Implementation of AI-enabled POCUS has been rolled out in staggered fashion, two facilities at a time starting with the health centers in Chongwe and Kafue. Because of the complexity of integrating AI-enabled POCUS into hospital settings, the two Lusaka sites were part of the third phase.

**Table 5:** Study facilities and associated characteristics.

District	Facility*	Designation	New ANC visits in 2024
Chongwe District	A	Rural health center	2,389
	B	Rural health center	290
Kafue District	C	Rural health center	334
	D	Urban health center	618
Lusaka District	E	Level 1 hospital (urban)	4,596
	F	Level 1 hospital (urban)	5,514

* Facilities anonymized.

At the start of the pilot program, sites in Chongwe and Kafue Districts did not have on-site access to obstetric ultrasound services. Instead, referrals were made to the district hospital or other providers, including the private sector. In Lusaka district, both facilities were Level 1 hospitals, where ultrasound services are available only by referral. Because these services are outside of antenatal care, access is often limited and delayed.

### Data management

The study has four primary sources of data: (1) audio recordings from FGDs and IDIs, transferred to a secure, centralized server; (2) information obtained from the patient and referral registers; (3) observational data obtained from TMS and ISA; and (4) patient exit survey results. These data are queried, curated, and cleaned as needed. All study computers and electronic devices are password-protected and their access restricted to authorized study personnel. All study audio-recording devices and study-specific paper forms are stored securely at the study site headquarters in locked filing cabinets in a locked room.

### Ethical considerations

The study protocol was approved by the University of North Carolina at Chapel Hill Institutional Review Board (Chapel Hill, NC, USA), the University of Zambia Biomedical Research Ethics Committee (Lusaka, Zambia), and the Zambia Ministry of Health National Health Research Authority prior to initiation. Written informed consent if provided for all focus group discussion and in-depth interview participants. Verbal informed consent is provided for all time motion study, patient exit surveys, and implementation strategy assessment participants.

### Dissemination of findings

All reports and publications of collected data will be presented in aggregate form only omitting all names or other identifiers of participating individuals. Comprehensive results will be disseminated to the participating facilities, key stakeholders, including government partners, and the Ministry of Health. Results will also be presented at national, regional, and international meetings, and submitted to international peer-reviewed journals. Findings will be made available through appropriate national and international channels, including academic and public health research symposia.

## Discussion

Introduction of portable POCUS probes and AI-enabled tools into routine ANC can significantly expand access to obstetric ultrasound services by reducing equipment needs, training costs, and provider and patient time burden. Successful implementation has the potential to improve pregnancy dating, especially for underserved populations in areas where trained sonographers are scarce. To date, such experiences are limited and this represents a gap in the field.

Despite the potential clinical and public health gains from AI-enabled ultrasound [24], GA estimation represents one of the only models to be rigorously evaluated and approved for clinical use in antenatal settings [13,14]. In places like Zambia, where obstetric ultrasound is limited, the confirmation (or correction) of gestational age can have important downstream effects on clinical decision-making and management. At the same time, we recognize that the diagnostic capacity for this intervention is relatively narrow and does not represent the full scope of obstetric sonography. But the successful introduction of this AI-enabled POCUS—even with this single feature—paves new ground and can provide a valuable foundation as the portfolio of AI tools for obstetric ultrasound expands.

To our knowledge, this protocol is among the first evaluate an integrated package that combines portable and rechargeable equipment (i.e., POCUS probes and tablets), basic ultrasound training (i.e., fetal heart rate detection), and an AI-enabled tool to estimate GA. Like others [25], we sought to implement this package in the African region—in this case, Zambia—because of the outsized need for such capacity and the operational challenges to more conventional obstetric ultrasound delivery models. These perceptions have been largely affirmed by the Zambian Ministry of Health, which has supported the PIKABU program at the central, provincial, and district levels.

Our evaluation is designed to potentially inform broader implementation. By taking an implementation science approach, we envision key evaluation findings that can support efforts in other resource-constrained settings globally. We employ several evaluation components to better understand acceptability, feasibility, and fidelity of the intervention package. Although AI-enabled technologies are becoming increasingly common, there may be individual, cultural, and community factors that may affect their uptake. In a study of African providers, researchers, and implementors, for example, respondents raised concerns about algorithm accuracy, data privacy, and ownership [24]. Our mixed methods approach provides perspectives from patients and providers alike and adds depth to our investigation. Documentation of implementation strategies offers valuable insights about how to address health systems challenges.

There are many strengths of this implementation evaluation. The CFIR was integrated throughout the research process, informing the design of multiple data collection tools and structuring qualitative data analysis, strengthening the applicability of findings [26]. The iterative nature of the evaluation and feedback to the facilities allows for real-time review and modification of the implementation process when appropriate. Finally, multiple facilities representing a diverse array of ANC setting with unique contextual factors that may serve as barriers or facilitators to the implementation enhance the generalizability of the findings.

We also recognize a few potential limitations. First, biases inherent to data gathered via focus group discussions, in-depth interviews, exit surveys and observations include moderator, observer, social-desirability, dominance, and conformity [27]. All study members conducting evaluation components undergo training informed by an expert in the field of qualitative data analysis and observational data collection in an attempt to mitigate these biases. Second, data collected from pre-existing clinical records and registers may be limited as these instruments were not designed for programmatic purposes. ANC cards and registers were reviewed prior to developing corresponding evaluation components to maximize the exaction of available data which would inform the study outcomes. Third, challenging clinical contexts such as high staff turnover and a reliance on voluntary and temporary staff pose potential challenges to the sustainability of implementation; however, this is the reality of many ANC facilities in these and similar settings.

In summary, we describe an evaluation protocol to understand the acceptability, feasibility, and fidelity of AI-enabled POCUS implementation in Zambia’s Lusaka Province. The multifaceted approach will generate evidence for policy makers, district and health facility managers, and healthcare providers, with the ultimate aim of providing real-world evidence to support future implementation.

## Supporting information

S1 File Inclusivity in global research questionnaire for PLOS journals. (DOCX)
